# Metabolic Features and Nutritional Interventions in Chronic Diseases

**DOI:** 10.3390/nu17111826

**Published:** 2025-05-28

**Authors:** Yongting Luo, Peng An, Junjie Luo

**Affiliations:** Department of Nutrition and Health, China Agricultural University, Beijing 100193, China; luo.yongting@cau.edu.cn (Y.L.); an-peng@cau.edu.cn (P.A.)

Metabolic dysfunction is closely linked to the pathogenesis and progression of various chronic diseases [1], including aging, cancer, and disorders of the cardiovascular system, liver, skin, and gut. Nutritional interventions, utilizing bioactive compounds or tailored dietary regimens, have emerged as effective strategies to modulate metabolic dysfunctions [2], thereby mitigating disease progression and reducing incidence rates. This Special Issue of *Nutrients*, entitled *“Metabolic Features and Nutritional Interventions in Chronic Diseases”*, serves as an interdisciplinary platform to compile research on metabolic characterization in chronic diseases and the development of novel nutritional interventions aimed at improving disease outcomes through metabolic regulation.

This Special Issue comprises ten papers, including seven research articles, one clinical trial, one secondary analysis of a clinical trial, and one retrospective study. The topics covered include the following: the potential association of serum vitamin B6 levels with preoperative non-small-cell lung cancer (NSCLC) upstaging (one paper); a clinical trial on preoperative multistrain probiotic supplementation on bariatric treatment outcomes (one paper); the therapeutic effect of Epigallocatechin-3-gallate (EGCG), a tea-derived antioxidant, on DSS-induced colitis and its underlying metabolic mechanisms (one paper); exploring the potential of a naturally occurring metabolite, 3-hydroxybutyrate (3HB), as an anti-aging intervention in two aging models (one paper); the experimental evaluation of caprylic acid and eicosapentaenoic acid on lipids and inflammatory levels, as well as their underlying mechanisms (one paper); the identification of a low-zinc diet as a potential nutritional intervention approach for the prevention of thoracic aortic dissection (TAD) through the modulation of aortic inflammation (one paper); exploring celastrol-enriched peanuts in reducing atherosclerosis and obesity (one paper); the potential beneficial effect of probiotics in reducing cardiovascular risk and depression (one paper); and nutritional interventions for non-alcoholic fatty liver disease (NAFLD) using either pea albumin or hawthorn ethanol extract (two papers).

NAFLD is a major liver disease worldwide, with a global adult prevalence of nearly 30% [3]. It is characterized by excessive lipid deposition in hepatocytes and can progress from simple fat accumulation to steatohepatitis, fibrosis, cirrhosis, and even hepatocellular carcinoma [4]. Multiple factors contribute to the pathogenesis of NAFLD [5], such as excessive dietary fat intake, abnormal hepatic lipid metabolism, and imbalanced gut microbiota. For this reason, identifying potential nutritional interventions that regulate the altered lipid metabolism in the liver is crucial for the prevention and management of NAFLD. In this Special Issue, Shucheng Zhang et al. reported that the oral administration of pea albumin (PA), the major protein extracted from pea (*Pisum sativum* L.) seeds, effectively ameliorated high-fat-diet-induced NAFLD in mice (Contribution 1). The intervention with PA lowered serum cholesterols, reduced hepatic steatosis and lipid accumulation, with concomitant improved insulin resistance, and reduced hepatic oxidative stress and inflammatory responses. In a second study on NAFLD within this Special Issue, Tianyu Wang et al. demonstrated that hawthorn ethanol extract (HEE) could effectively diminish hepatic lipid accumulation through facilitating triglyceride breakdown and suppressing fatty acid synthesis while also reducing blood lipids and liver inflammation (Contribution 2). The two studies highlight PA and HEE as promising dietary supplements for NAFLD management.

Besides NAFLD, metabolism dysfunction is also observed in many cardiovascular diseases [6], including atherosclerosis and aortic dissection. Alteration in lipid metabolism results in hyperlipidemia, an important contributor to atherosclerosis. Atherosclerosis has been considered a non-resolving autoinflammatory disease of the arterial walls [7]. As the main contributor of atherosclerotic cardiovascular disease (ASCVD), such as myocardial infarction and stroke, atherosclerosis is one of the leading causes of death worldwide [8]. Nutritional intervention has been considered a promising strategy for atherosclerosis [9]. In this Special Issue, Jiaxin Shi et al. innovatively developed a novel peanut cultivar enriched with celastrol (cel-peanut), a pentacyclic triterpenoid active component, and evaluated the anti-atherosclerosis effects (Contribution 3). They demonstrated that celastrol-enriched peanuts effectively normalized blood lipid profile, alleviated aortic plaque burden, reduced body weight, and promoted intestinal health in high-fat-diet-induced ApoE^−/−^ mice. These beneficial effects of celastrol-enriched peanuts might result from the anti-inflammatory, antioxidant, and immunomodulatory properties of celastrol. In parallel with this study, another study in this Special Issue evaluated the effect of fatty acids on the development of atherosclerosis. Xinsheng Zhang et al. investigated the effects of caprylic acid (C8:0) and eicosapentaenoic acid (EPA) on lipids, inflammatory levels, and the JAK2/STAT3 pathway in ABCA1-deficient mice and macrophages (Contribution 4). They observed that EPA had better effects than C8:0 on inhibiting inflammation and improving blood lipids in the absence of ABCA1, and upregulation of the ABCA1 expression pathway by functional nutrients may provide potential targets for the prevention and treatment of atherosclerosis. On the other hand, by performing a secondary analysis of a randomized clinical trial, Oliwia Gawlik-Kotelnicka et al. demonstrated that probiotics used along with a healthy diet may provide additional benefits by reducing cardiovascular risk (Contribution 5). However, Marta Potrykus et al. reported a randomized, double-blind, placebo-controlled clinical trial, where they observed that preoperative multistrain probiotic supplementation showed no apparent effect on body weight changes or cardiometabolic risk factors in bariatrics (a surgical treatment of obesity) (Contribution 6). Therefore, although microbiota plays a crucial role in the development of and therapeutic options in obesity, the role of probiotic supplementation as a nutritional intervention in bariatric surgery requires further investigation. In addition to atherosclerosis, nutritional intervention also shows promise in the management of aortic dissection, a life-threatening condition caused by a tear in the intimal layer of the aortic wall. By employing a classical-aminopropionitrile monofumarate (BAPN)-induced aortic dissection model in mice, Lin Zhu et al. demonstrated that low zinc could improve aortic dissection and rupture and reduce mortality through attenuating aortic inflammation and suppressing the phenotype switch of aortic smooth muscle cells from contractile to synthetic types (Contribution 7). This study suggested that low zinc may serve as a potential nutritional intervention approach for the prevention of aortic dissection.

Nutritional intervention also shows promise in fighting other diseases or disease complications, such as cancer [10], tissue inflammation [11], and aging [12]. In this Special Issue, Lu Liu et al. evaluated a possible relationship between vitamin B levels and the development and progression of lung cancer (Contribution 8). Their retrospective study established a positive association of serum vitamin B6 levels with the intrapulmonary lymph node and/or local pleural metastases of non-small-cell lung cancer, and this association was stronger in females, current smokers, current drinkers, and those with a family history of cancer. Furthermore, Junzhou Chen et al. found that supplementation with a tea-derived antioxidant called EGCG could effectively alleviate DSS-induced colitis in mice by inhibiting ferroptosis and reducing oxidative damage in colonic epithelial cells (Contribution 9). Finally, Yongpan An et al. found that the supplementation of 3-hydroxybutyrate (3HB), an endogenous metabolite with established safety, effectively delayed cellular senescence and extended lifespan in both yeast and mice via metabolic reprogramming (Contribution 10). This study highlights 3HB as a potential anti-aging nutritional intervention.

In summary, chronic diseases exhibit distinct metabolic dysfunctions, and nutritional interventions—whether through bioactive nutrients or dietary modifications—offer promising avenues for disease management. The studies in this Special Issue underscore the therapeutic potential of nutritional strategies in modulating metabolic pathways, oxidative stress, and immune responses. These findings reinforce the paradigm of “food as medicine” and highlight the need for the further clinical exploration of these nutritional interventions.
